# Metabolic perturbations and key pathways associated with the bacteriostatic activity of *Clitoria ternatea* flower anthocyanin fraction against *Escherichia coli*

**DOI:** 10.1099/acmi.0.000535.v5

**Published:** 2023-06-28

**Authors:** Ethel Jeyaseela Jeyaraj, Mei-Ling Han, Jian Li, Wee Sim Choo

**Affiliations:** ^1^​ School of Science, Monash University Malaysia, Jalan Lagoon Selatan, 47500 Bandar Sunway, Selangor, Malaysia; ^2^​ Monash Biomedicine Discovery Institute, Department of Microbiology, Monash University, Clayton, Victoria, Australia

**Keywords:** acylated anthocyanin, antimicrobial, blue pea flower, infectious diseases, metabolomics, natural product

## Abstract

*Clitoria ternatea* flowers are rich in anthocyanins and possess various biological activities. Specifically, the antibacterial mechanism of action of *C. ternatea* anthocyanins remains unknown and was investigated in *Escherichia coli *. A time–kill assay was used to assess the antibacterial activity and the metabolic perturbations in *E. coli* were investigated utilizing liquid chromatography–mass spectrometry (LC-MS)-based metabolomics. Pathway analyses were carried out for metabolites showing ≥2-fold changes. The anthocyanin fraction remarkably reduced the growth of *E. coli* at 4 h by 95.8 and 99.9 % at minimum inhibitory concentration (MIC) and 2× MIC, respectively. The anthocyanin fraction (MIC) had a bacteriostatic effect and was shown to have perturbed glycerophospholipids (1-acyl-sn-glycero-3-phosphoethanolamine, phosphatidylglycerol, diacylglycerol and cardiolipin), amino acids (valine, tyrosine and isoleucine) and energy (ubiquinone and NAD) metabolites at 1 and 4 h. This study demonstrated significant metabolic perturbations of the glycerophospholipid, amino acid and energy metabolism, with these being the key pathways involved in the bacteriostatic activity of anthocyanins from *C. ternatea*, which may have promise as bacteriostatic agents for *E. coli *-related infections.

## Data Summary

All data is available at https://doi.org/10.26180/21687398.v1 [1].

## Introduction

Although *Escherichia coli *, Gram-negative bacteria of the family *Enterobacteriaceae *, comprise a common, normal part of the flora in the human gut, they have become a concern because of their pathogenic strains, which are known to cause severe infections, along with the increase in the emergence of multidrug resistance globally [2]. *E. coli* have been identified as significant causative pathogens in bloodstream and urinary tract infections worldwide in community and healthcare settings [3]. They have displayed high rates of resistance to antibiotics such as aminoglycosides, aminopenicillins, fluoroquinolones and third-generation cephalosporins, further complicating and limiting treatment options [3].

The rise in antimicrobial resistance means there is a need for new antimicrobial agents or alternative therapeutic methods/strategies to target infectious diseases. Plants are a rich source of compounds with diverse functional groups in which many are yet to be explored for their therapeutic potential [4]. The *Clitoria ternatea* (‘blue pea’ or ‘butterfly pea’) plant belongs to the family *Fabaceae* and is known to grow in various countries in Asia and South and Central America [5, 6]. The leaves, flowers and roots of this plant have been used since ancient times to treat a variety of ailments and health conditions besides reports on their various biological activities (e.g. antioxidant, antidiabetic, anti-inflammatory, antifungal and antiproliferative/anticancer) [5, 6]. *C. ternatea* flowers have ornamental value and are also used as a food colouring agent due to the presence of the compounds known as anthocyanins [7]. The crude extract of the flower has been reported to show antibacterial activity against various bacterial strains (e.g. *E. coli *, *Klebsiella pneumoniae *, *Bacillus subtilis* and *Staphylococcus aureus *), which has mainly been attributed to its major compounds, flavonols and anthocyanins [8, 9].

Metabolomics is a comprehensive analysis investigating large-scale metabolite levels in biological systems. It can be further categorized as targeted (identifies and quantifies selected metabolites of interest, e.g. enzymes, substrates or compounds of a particular pathway) or untargeted (involves measuring all metabolites in a desired biological experimental system, which usually generates a new hypothesis based on the outcome) approach, depending on the nature of the study [10, 11]. Metabolomics is increasingly being used to elucidate the mechanism of action of drugs in a biological system for drug discovery and development [12]. Our previous study [13] obtained an anthocyanin fraction from this flower to determine the antibacterial activity against various pathogenic bacterial strains. Antibacterial activity was observed against *B. subtilis *, *E. coli* and *Bacillus cereus *. As *E. coli* is a pathogen of importance, the present study aimed to determine the antibacterial activity and the mechanism of action of the anthocyanin fraction against *E. coli* through a metabolomics study.

## Methods

### Preparation and extraction of samples

*Clitoria ternatea* cv. Double Blue flowers (freshly harvested) were procured from a horticultural nursery in Subang Jaya, Malaysia. *C. ternatea* flower petals were plucked carefully for use in the study, while the remaining parts were discarded. Before extraction, the petals were cut into smaller pieces. Ethanol extracts of the fresh flower material were prepared by immersing the flowers in 50 % ethanol with a solid to liquid ratio of 1 : 20 (g ml^−1^) for 3 h at room temperature (25 °C) with constant shaking. Extracts were then vacuum-filtered, and a rotary evaporator was used to concentrate the solution under vacuum at 45 °C. The concentrated solution was freeze-dried and stored at −80 °C until further analysis.

### Semi-purification of crude extract by Amberlite XAD-16 column chromatography

The anthocyanin fraction was obtained according to the method in a previous study [14]. Distilled water (100 ml) was used to dissolve the freeze-dried extract of *C. ternatea* flowers (5 g), adjusted to pH 2 using 1M HCl followed by partitioning with ethyl acetate (100 ml) to facilitate the removal of flavonols. The aqueous fraction (containing anthocyanins) was collected and again partitioned two more times with ethyl acetate. The aqueous fraction (containing anthocyanins) was concentrated to a volume of 10 ml under vacuum at 37 °C in a rotary evaporator. The aqueous fraction (containing anthocyanins) was subjected to further purification using Amberlite XAD-16 column chromatography. A chromatography column [S24/29, 25(D)×300 mm (l)] with a sintered glass disc (porosity 0) and a stopcock with a PTFE key were used for the open column chromatography. The Amberlite XAD-16 adsorbent was immersed in methanol overnight prior to loading to ¾ of the column height. Briefly, 1 l of purified water was used to rinse the column followed by activation with 0.5 l of aqueous sodium hydroxide (2%) solution. Acidified water (1 l) was used to wash the column to condition it to pH 3. The concentrated aqueous fraction (10 ml) of the anthocyanin fraction was loaded onto the column, which was then rinsed with acidified water (0.3 l at pH 3) to remove phenolic acids at a flow rate of 10 ml min^−1^. Anthocyanins were then eluted with acidified methanol [95 : 5, methanol : acidified water (pH 2), v/v]. The methanol (contains anthocyanins) fraction was freeze-dried and stored at −80 °C until further analysis. The extraction yield of the anthocyanin fraction using Amberlite XAD-16 column chromatography was 18.2 %, as reported in our previous study [13].

### Time–kill assay of *E. coli* ATCC 25922

The culture of *E. coli* ATCC 25922 was prepared from frozen stock (−80 °C) on tryptic soy agar plates and incubated at 37 °C for 18 h. A single colony was obtained and inoculated into brain heart infusion (BHI) broth (10 ml), incubated at 37 °C for 18 h in a shaking water bath (180 r.p.m.). The overnight culture diluted with BHI broth (1 : 100) was grown to obtain an OD_600nm_ of 0.50 to achieve an early logarithmic growth phase (i.e. ~10^8^ c.f.u. ml^−1^), followed by dilution (1 : 100) in 20 ml of BHI broth alone (control group) and BHI broth containing *C. ternatea* anthocyanin fraction at ½ MIC (5 mg ml^−1^), MIC (10 mg ml^−1^) and 2× MIC (20 mg ml^−1^) for 1, 4 and 24 h. One millilitre of sample was taken at 0, 1, 4 and 24 h and serial dilutions were performed according to the pour plate method as described below to determine bacterial growth (c.f.u. ml^−1^). The formula below was used to count the percentage reduction in the total viable count of c.f.u.:

$$Percentage\;reduction=\frac{Count\;of\;untreated\;group\;at\;x\;interval\;-\;count\;of\;treated\;group\;at\;x\;interval}{Count\;of\;untreated\;group\;at\;x\;interval}\times 100$$

#### Growth curve (pour plate method)

The effect of the anthocyanin fraction at ½ MIC (5 mg ml^−1^), MIC (10 mg ml^−1^) and 2× MIC (20 mg ml^−1^) for 1, 4 and 24 h on *E. coli* ATCC 25922 growth was evaluated using the standard pour plate method [15]. Serial dilutions (1 : 10) at 10^3^ to 10^6^ of treated bacteria culture were performed and 100 µl of individual diluted bacteria culture was dispensed onto the bottom of Petri dishes. Then 18 ml of molten Mueller–Hinton agar (45 °C) was poured and mixed with the sample by gently swirling the plate. The plates were incubated at 37 °C for 24 h after the agar solidified. The number of *E. coli* ATCC 25922 colonies was recorded from plates with 30–300 colonies as c.f.u. The microbial data are presented as log c.f.u. ml^−1^ [16].

### Statistical analysis

The experiments above were performed in independent triplicates and the results were expressed as the mean value±standard deviation. The data were analysed using one-way analysis of variance (ANOVA) followed by post-hoc Tukey’s test to analyse the statistical difference exhibited by *E. coli* ATCC 25922 in terms of growth in untreated (control) and treated groups [½ MIC (5 mg ml^−1^), MIC (10 mg ml^−1^) and 2× MIC (20 mg ml^−1^)]. The significance was set at *P*<0.05 using SPSS 23 software (IBM, New York, USA).

### Bacterial culture preparation for metabolome analysis

Bacterial isolates of *E. coli* ATCC 25922 were sub-cultured from −80 °C stocks onto tryptic soy agar. A single colony was obtained and inoculated in BHI broth (10 ml). It was incubated overnight at 37 °C with constant shaking (180 r.p.m.). The overnight culture was diluted by 1 : 100 (2.5 ml was inoculated into 500 ml) of BHI broth and grown to obtain an OD_600_ of 0.5 (mid-exponential growth phase) at 37 °C with constant shaking (180 r.p.m.). Subsequently, 20 ml of mid-exponential culture (OD_600_ = 0.5) was collected into a 50 ml Falcon tube (this was used as 0 h). For each sample, 50 ml of the mid-exponential culture was treated with the anthocyanin fraction of *C. ternatea* (AF) resuspended in BHI broth at MIC (10 mg ml^−1^), while the untreated group served as a control sample. Three biological replicates of each group were prepared. The bacterial cultures (20 ml) of treated and untreated groups were collected at 1 and 4 h.

### Extraction and liquid chromatography–mass spectrometry (LC-MS) analysis of cellular metabolites

Cellular metabolites were extracted using a previously reported method [17, 18]. The bacterial cultures (20 ml) of treated and untreated groups at 0, 1 and 4 h were immediately transferred into ice-cold 50 ml Falcon tubes. To stop the metabolic processes, the samples were quenched rapidly in a dry ice/ethanol bath for 30 s. The culture was then normalized to 0.5 at OD_600_ (cell counts were at 10^8^ c.f.u. ml^−1^) using fresh BHI broth. The normalized culture (10 ml) was centrifuged (3220 ***g*** at 4 °C for 10 min) and the cell pellet was obtained. Ice-cold 0.9 % sodium chloride (2 ml) was used to wash the cell pellet twice followed by centrifugation (3220 ***g*** at 4 °C for 5 min). The residue after centrifugation was removed. After discarding the supernatant, 0.5 ml of dry-ice cold chloroform : methanol : water (1 : 2 : 0.8, v/v) extraction solvent containing internal standard at 1 µM of 2-amino-2-(hydroxymethyl)propane-1,3-diol (TRIS) was added to resuspend the cells and facilitate the extraction of the intracellular metabolites. Liquid nitrogen was used to freeze the samples and allowed to thaw on ice to promote the release of intracellular metabolites. The freeze–thaw process was performed thrice and followed by centrifugation (3220 ***g*** at 4 °C for 10 min). The supernatant was obtained and transferred to 1.5 ml centrifuge tubes and centrifuged (14400 ***g*** at 4 °C for 10 min). The samples were stored at −80 °C prior to LC-MS analysis. The samples (3 µl) were analysed on a 1290 Infinity LC system coupled to a 6520 Accurate-Mass Q-TOF mass spectrometer with a dual ESI source (Agilent, Santa Clara, CA, USA) coupled to a HILIC-Z column (2.7 µm, polymeric, 100×2.1 mm; Infinitylab Poroshell, Agilent). The run was performed in positive and negative ion modes at a resolution of 35 000. The detection range was 85 to 1275 *m/z*. The mobile phases were 20 mM ammonium acetate (A) and acetonitrile (B) at a flow rate of 0.3 ml min^−1^. The applied condition was as follows: 20 % of solvent A and 80 % B to 50 % B over 15 min, and to 5 % B at 18 min. The system was washed with 95 % A and 5 % B for 3 min followed by 20 % A and 80 % B for 8 min to re-equilibrate the column. Nitrogen was used as the desolvation gas, at 300 °C and a flow rate of 60 l h^−1^, and He gas was used as the damping gas, with a declustering potential of 40 eV, a collision energy of 5 eV and a collision cell entrance potential of 10 eV. The samples were run in a single batch of LC-MS to avoid batch-to-batch variability [19].

### Data processing, bioinformatics and statistical analysis

MZmine 3 was used to analyse raw LC-MS data for chromatographic peak detection. Metabolites were putatively identified by comparing the *m/z* values and molecular formula with the online databases *E. coli* Metabolome Database (ECMDB: https://ecmdb.ca/), Kyoto Encyclopedia of Genes and Genomes (KEGG, https://www.genome.jp/kegg/) and LIPID MAPS (https://www.lipidmaps.org/) [20–22]. The peak height was used to quantify the amount of each metabolite and was normalized by the median and log-transformed utilizing MetaboAnalyst 5.0. Principal component analysis (PCA) was applied to the treated and untreated groups. Univariate statistical analysis was performed using Welch’s *t*-test (*P*<0.05) to identify significant changes between groups. Metabolites that were significant and had ≥2-fold change (FC) (i.e. log_2_ FC ≥1 or ≤1) were further analysed for metabolic changes between treated and untreated groups. Pathway analysis of significantly perturbed metabolites was carried out using ECMDB, KEGG and LIPID MAPS [20–22].

## Results and discussion

### Antibacterial activity of the *C. ternatea* anthocyanin fraction against *E. coli* ATCC 25922

**Fig. 1.**
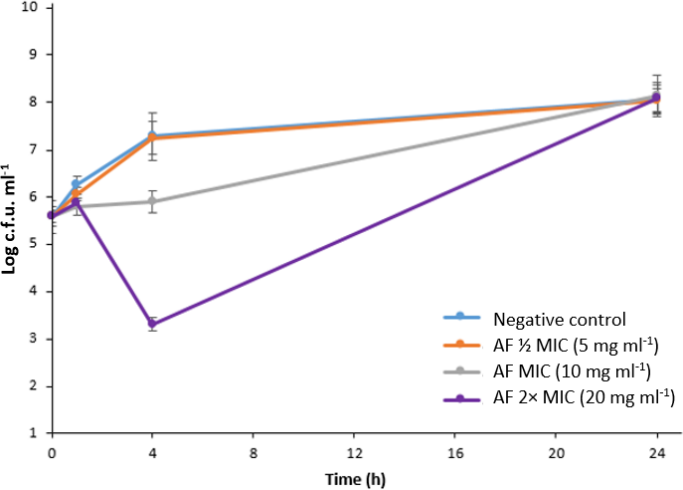
Time–kill assay. Log c.f.u. ml^−1^ of *E. coli* ATCC 25922 for 1, 4 and 24 h treated with *C. ternatea* anthocyanin fraction (AF) at ½ MIC (5 mg ml^−1^), MIC (10 mg ml^−1^) and 2× MIC (20 mg ml^−1^). The standard deviations of log c.f.u. ml^−1^ of three biological replicates are represented by the error bars (*P*<0.05).

The antimicrobial activity of *C. ternatea* anthocyanin fraction against a laboratory strain *E. coli* ATCC 25922 utilizing the agar dilution method (ADM) showed the MIC value to be 10 mg ml^−1^ at 24 h in our previous study. The MIC is defined as the lowest concentration of an antimicrobial that will inhibit the visible growth of a micro-organism after overnight incubation [13]. The concentration of the fraction was found not to affect the viability of *E. coli* at ≤5 mg ml^−1^ (viability >95 %). A time–kill assay was performed with *E. coli* treated at ½ MIC (5 mg ml^−1^), MIC (10 mg ml^−1^) and 2× MIC values (20 mg ml^−1^) of *C. ternatea* anthocyanin fraction (Fig. 1). The time–kill assay showed that the fraction at ½ MIC value did not affect the bacterial growth over a period of 24 h. The reduction of bacterial growth (c.f.u. ml^−1^) by treatment groups was determined at respective time points compared to the untreated group. At 1 h, the bacterial growth (c.f.u. ml^−1^) was 11.2×10^5^, 6.0×10^5^ and 7.4×10^5^ at ½ MIC, MIC and 2× MIC, respectively, compared to the untreated control, which was 17.6×10^5^ c.f.u. ml^−1^. While at 4 h, the bacterial growth (c.f.u. ml^−1^) was 17.4×10^6^, 8.0×10^5^ and 2.0×10^3^ at ½ MIC, MIC and 2× MIC, respectively, compared to the untreated control, which was 19.0×10^6^ c.f.u. ml^−1^. The highest reduction of bacterial growth (c.f.u. ml^−1^) by the treatment group was observed at 4 h, with 95.8 and 99.9% reduction at MIC and 2× MIC, respectively (Fig. 1).

Our previous study and another group of researchers found that the crude flower extract (up to 50 mg ml^−1^) did not have antibacterial activity against *E. coli* [8, 13]. However, it was different to that reported by a study that showed the crude extract to have an MIC value of 1.25–5.0 mg ml^−1^ (broth dilution method) against *E. coli *, while another study reported an activity at 100 mg ml^−1^ in a disc diffusion assay [9, 23].

The impact of regional differences of the compound composition of the *C. ternatea* crude flower extract is unknown, as the composition was not reported in these studies. The crude flower extract is known to have a much lower content of anthocyanins, suggesting that the high content of anthocyanins (composed mainly of ternatin B2/B3, ternatin D1 and ternatin D2) in the fraction used in this study is responsible for the antibacterial activity. This can also be supported by the findings observed in *B. cereus* and *B. subtilis* strains, where the MIC of the anthocyanin extract was 16 times lower than the MIC of the crude extract [13].

The anthocyanin composition in the fraction is based on our previous study, which determined the composition of anthocyanins (%) via LC-MS analysis in the anthocyanin fraction obtained from Amberlite XAD-16 open column chromatography [13]. Ternatin B2, ternatin D1 and ternatin D2 were the most abundant anthocyanins, with a peak area of 23.9, 20.4 and 20.0 %. Other anthocyanins, such as ternatin B3, ternatin B4, ternatin C1, ternatin C2 and ternatin D3 were also detected and reported by other studies [13, 24].

The MIC (10 mg ml^−1^) of the *C. ternatea* anthocyanin fraction was determined in our previous study using the agar dilution method [13]. However, in this current study, the time–kill study was performed using the suspension method (in a flask with large volume as the metabolomics study requires a larger amount of culture to proceed with study analysis) with constant shaking, which may further accelerate the growth of bacterial culture, contributing to the effect observed at 24 h in the current study.

In this current study, the *C. ternatea* anthocyanin fraction at MIC and 2×MIC value at 4 h displayed a bacteriostatic effect, as the reduction of log c.f.u. ml^−1^ of *E. coli* ATCC 25922 (Fig. 1) at 4 h for MIC or 2× MIC was not greater than a 3 log_10_-fold decrease in c.f.u. [25], which shows that not all bacterial cells are killed at 4 h and the bacteriostatic effect is not maintained at 24 h. A similar pattern has also been observed in another study with other natural compounds from garlic and guava [26]. It is also suggested that the antibacterial activity exhibited by *C. ternatea* anthocyanin fraction may be contributed by the synergistic activity of the anthocyanins in the fraction.

Bacteriostatic agents can be defined as agents that are able to prevent the growth of bacteria without causing bacterial cell death directly [27, 28]. Drugs with a bacteriostatic effect are more advantageous than those that are bactericidal as they may help to prevent drug resistance [27–29]. Observations have shown bacteriostatic agents to cause a lower incidence of toxic shock and more tolerable side effects clinically [27]. Apart from that, from our previous study [13], although the crude extracts of *C. ternatea* flower displayed toxicity at doses above 156.3 µg ml^−1^ against HEK-293 cells (human embryonic kidney cells), the findings from the acute toxicity study in rats show the extract to be rather safe for consumption.

An acute toxicity study was performed using albino Wistar rats, which were treated orally with 50 % ethanol extract of *C. ternatea* flowers at 2000 mg kg^−1^ body weight. The treatment group showed no signs of mortality or abnormality and there was no significant difference in the haematological values compared to the control untreated group, which indicates no acute toxicity of *C. ternatea* flower extracts up to 2000 mg kg^−1^ [30]. The anthocyanin fraction could potentially be used to reduce *E. coli *-related infections. However, further studies investigating the toxicity in preclinical models would be essential.

### Metabolic profile of *E. coli* ATCC 25922 treated with *C. ternatea* anthocyanin fraction

**Fig. 2.**
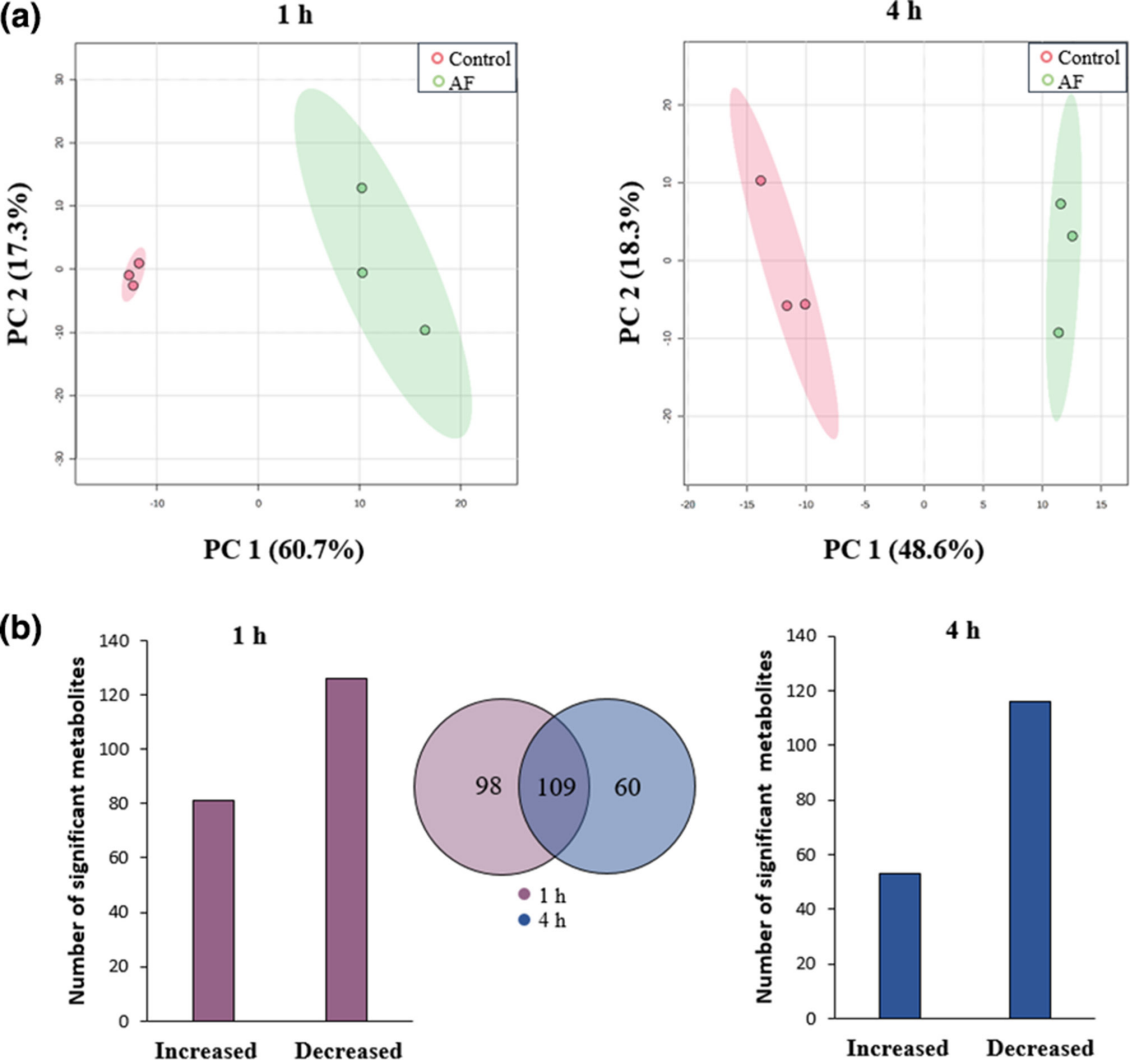
Metabolomic analyses of *E. coli* ATCC 25922 treated with *C. ternatea* flower anthocyanin fraction (AF) at 1 and 4 h. (**a**) The metabolic changes in *E. coli* with treatment with *C. ternatea* AF as indicated by principal component analysis (PCA) plots at 1 and 4 h from the control. (**b**) Bar charts and Venn diagram of significantly perturbed metabolites in *E. coli* treated with AF at 1 (purple) and 4 h (blue). Significantly perturbed metabolites were selected based on log_2_ FC ≤−1 or ≥1 and *P*<0.05.

**Fig. 3.**
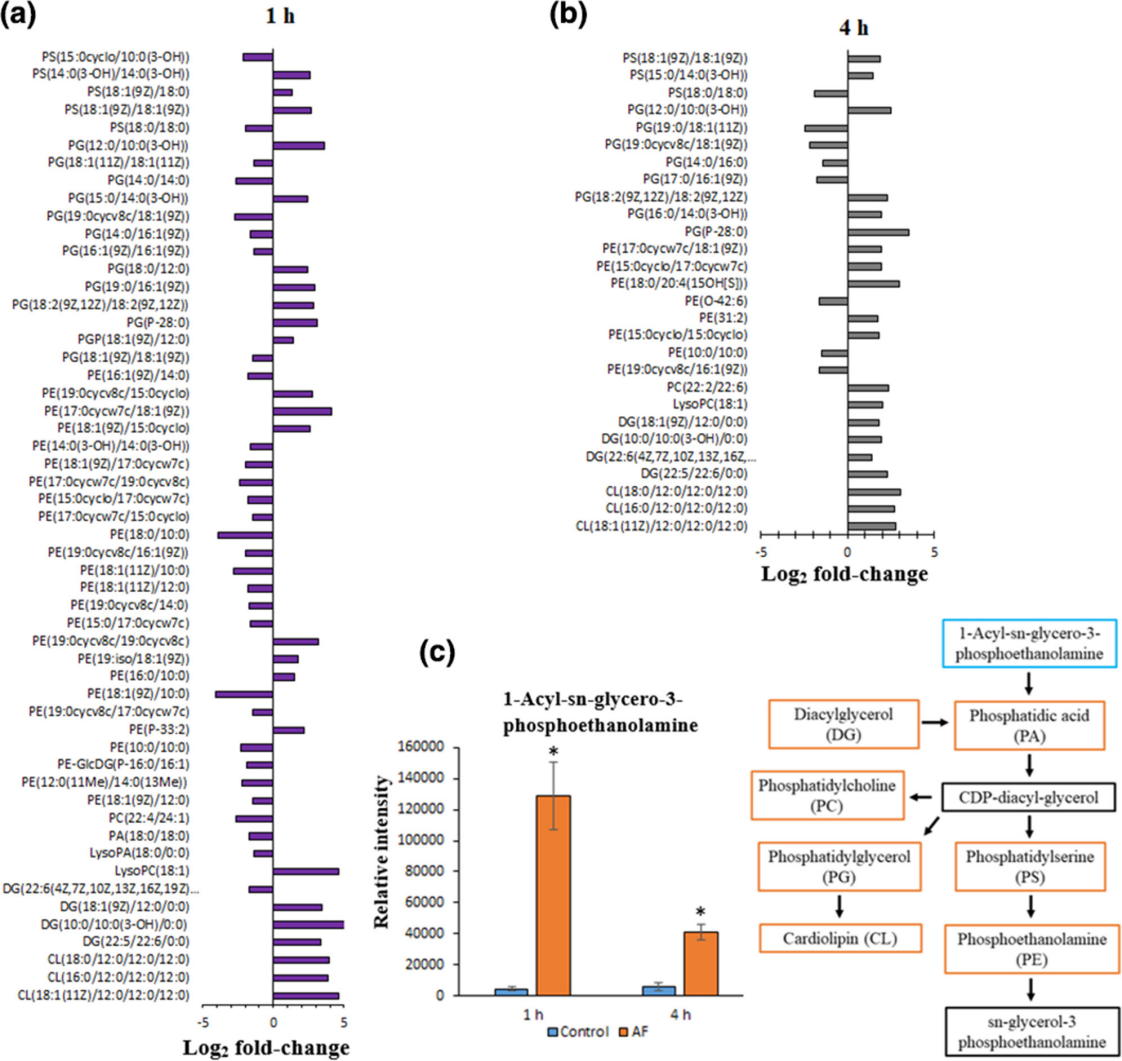
Metabolomic analysis of *E. coli* ATCC 25922 treated with *C. ternatea* flower anthocyanin fraction (AF) at 1 and 4 h. *E. coli* lipid perturbations upon treatment with AF at 1 (**a**) and 4 h (**b**). The schematic diagram and bar charts (**c**) show the significantly perturbed lipids in *E. coli* following AF treatment at 1 and 4 h of the metabolites in lipid biosynthesis pathways. Blue/orange in the schematic diagram or ‘*’ show significantly perturbed metabolites (log_2_ FC ≤1 or ≥1 and *P*<0.05).

The mechanism of action of the anthocyanin fraction of *C. ternatea* for its bacteriostatic activity against *E. coli* is not known and was further investigated utilizing untargeted metabolomics analysis to determine key signalling pathways that may be responsible for the antibacterial effect observed. Untargeted metabolomics was used, as it is not known which metabolites are or would be affected upon treatment with *C. ternatea* anthocyanin fraction. LC-MS-based metabolomics was used for analysing polar and nonpolar small molecules [31]. The metabolic perturbations of the *C. ternatea* anthocyanin fraction against *E. coli* were determined at its MIC (10 mg ml^−1^) at 1 and 4 h treatment (Fig. 2). Approximately 800 metabolites were detected in the control (untreated) and treated groups at 1 and 4 h and were subjected to PCA followed by statistical analysis utilizing the *t*-test. A clear clustering between treatment and control groups was observed for the metabolite profile at 1 (PC1=60.7 %) and 4 h (PC1=48.6 %) using PCA (Fig. 3a). The log_2_ fold change (≤−1 or ≥1) was determined for the metabolites that were significantly perturbed in the treatment group compared to the control. In total, the number of significantly affected metabolites at 1 h was 81/126 (up/down) and 53/116 (up/down) at 4 h. (Fig. 2b). These metabolites were then subjected to identification utilizing the online databases ECMDB, KEGG and LIPID MAPS followed by pathway analysis. The significantly perturbed metabolites were from the metabolism of lipids, amino acids and energy, which are further discussed below.

### Perturbation of phospholipid and glycerophospholipid metabolism by *C. ternatea* anthocyanin fraction

Glycerophospholipids are important components of the outer membrane (a dual membrane composed of lipopolysaccharide in the outer leaflet and glycerophospholipids in the inner leaflet) of Gram-negative bacteria, which is essential to protect the bacteria from toxic compounds and harsh environmental conditions. The inner membrane (symmetrical bilayer) of the bacteria, by contrast, is known to be composed of phospholipids, which are essential for the maintenance of barrier permeability and in support of its membrane proteins [32].

The glycerophospholipid molecules are mainly phosphatidylethanolamine (PE), followed by phosphatidylglycerol (PG), phosphatidylcholine (PC), phosphatidylserine (PS) and cardiolipin (CL). CL is synthesized in the inner membrane and transported to the outer membrane [33].

The treatment of *C. ternatea* anthocyanin fraction at 10 mg ml^−1^ (MIC) was found to have exerted growth inhibition and significantly perturbed the glycerophospholipid metabolites of *E. coli* at 1 and 4 h (Fig. 3). The levels of glycerophospholipids such as PE (18 : 0/10 : 0), PS [15 : 0cyclo/10 : 0(3-OH)], PG [19 : 0cycv8c/18 : 1(9Z)] and PE [18 : 1(11Z)/10 : 0] were decreased (log_2_ FC=−3.87 to −2.09), while 1-acyl-sn-glycero-3-phosphoethanolamine, PG [12 : 0/10 : 0(3-OH)], diacylglycerol (DG) (22 : 5/22 : 6/0 : 0) and CL (16 : 0/12 : 0/12 : 0/12 : 0) were increased (log_2_ FC=3.37 to 4.88) by *C. ternatea* anthocyanin fraction treatment at 1 h.

At 4 h, PS (18 : 0/18 : 0), PG [19 : 0/18 : 1(11Z)], PE (10 : 0/10 : 0) and PG [17 : 0/16 : 1(9Z)] were among those that decreased (log_2_ FC=−2.48 to −1.47), while 1-acyl-sn-glycero-3-phosphoethanolamine, CL (18 : 0/12 : 0/12 : 0/12 : 0), DG (22 : 5/22 : 6/0 : 0) and PE (31 : 2) were among those that increased (log_2_ FC=1.74 to 3.07).

A larger number of glycerophospholipid metabolites were perturbed at 1 h compared to 4 h (Fig. 3). The perturbations observed in the glycerophospholipid metabolites indicate membrane damage induced by *C. ternatea* anthocyanin fraction treatment. This may trigger enhanced synthesis and transport of glycerophospholipids to re-establish the outer membrane barrier of a bacterium [34]. This is also supported by the significant increase in the level of CL metabolites. CL metabolites play an essential role in remodelling of the bacterial outer membrane, as CL is also important for bacterial structural integrity and cell function [35].

*E. coli* membranes are mainly composed of PE (75%) followed by PG (20%) and CL (5%) [36, 37]. Changes in the environmental conditions or exposure to antibacterial agents can impose stress on bacteria, triggering certain responses to enable their survival and requiring major metabolic reprogramming [36]. The results obtained on the phosphoglycerolipid metabolite levels upon treatment with the anthocyanin fraction showed disruption of the optimum conditions required for membrane integrity, which may have caused alterations in cell physiology, which also compromises the integrity of the cell.

Compromised integrity of the cell membrane upon treatment could have led to a series of different cellular effects, such as cellular envelope structure modification, disruption of energy transduction metabolic pathways and impairment of membrane and macromolecule synthesis [37]. This can be supported by the findings reported in another study for the antibacterial effect of the anthocyanins of blueberries. The anthocyanins elicited bacterial cell membrane damage, resulting in leakage of intracellular materials as well as facilitating entry into the cell. It also further disrupted the tricarboxcylic acid (TCA) cycle and energy metabolism of the bacteria [38]. The results obtained in our study showed a remarkable effect of *C. ternatea* anthocyanin fraction, which triggered the perturbation of various phospholipids to cause disruption of membrane integrity to inhibit the growth of *E. coli *.

### Perturbation and inhibition of amino acid and energy metabolism of *C. ternatea* anthocyanin fraction

**Fig. 4.**
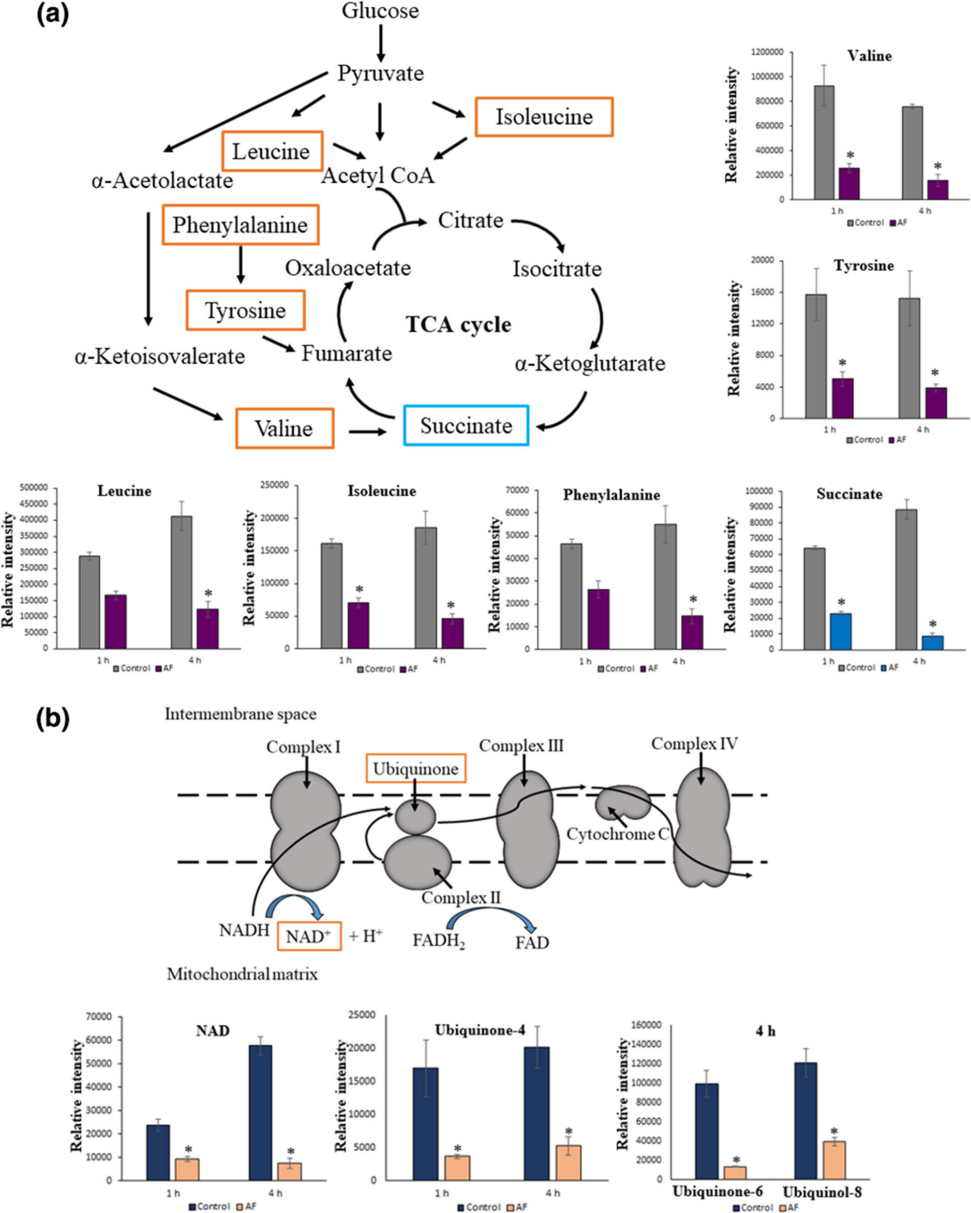
Metabolomic analyses of *E. coli* ATCC 25922 treated with *C. ternatea* flower anthocyanin fraction (AF) at 1 and 4 h. (**a**) Significant perturbations of amino acids and (**b**) energy metabolites in *E. coli* treated with AF at 1 and 4 h are represented in a schematic diagram and/or bar charts; ‘*’ indicates significantly perturbed metabolites (log_2_ FC ≤1 or ≥1 and *P*<0.05).

The disruption of bacterial membrane integrity by *C. ternatea* anthocyanin fraction could have facilitated its entry into the cell, affecting the stability of the intracellular environment and leading to the perturbations of the level of amino acid metabolites. The amino acids leucine (log_2_ FC=−1.04 and −1.74), isoleucine (log_2_ FC=−1.26 and −2.03), valine (log_2_ FC=−1.86 and −2.27), tyrosine (log_2_ FC=−1.64 and −1.96) and phenylalanine (log_2_ FC=−1.24 and −1.91) were reduced at both 1 and 4 h of treatment, respectively. There was a higher reduction of the metabolite levels of amino acids at 4 h compared to 1 h (Fig. 4a). The effect on the level of amino acid metabolites was higher later at 4 h as compared to the effect on glycerophospholipid metabolites, which was higher at 1 h (Fig. 3). This may be a subsequent effect that occurs after the perturbation of glycerophospholipids upon entry of anthocyanins into the cells.

Amino acids are the basic building blocks of proteins, and are also biomolecules that are essential in the biological functions of micro-organisms, such as metabolism, growth and survival of cells, formation of cell walls, division of cells and bacterial cell communication (quorum sensing). Enzymes, being composed of amino acids and classified as proteins, play a crucial role in facilitating the resistance of microbes to antimicrobial drugs [39]. The glycolytic pathway, the TCA cycle and the pentose phosphate pathway are oxidative pathways to obtain energy. The reduction of amino acid metabolite levels may reflect it being used as a substitute for carbon source due to the bacteriostatic effect of the anthocyanin fraction. This may be an adaptation mechanism of bacteria to thrive in environmental conditions to maximize their chance of survival [40, 41].

The TCA cycle is involved in the oxidation of different metabolic intermediates derived from the catabolism of lipids, carbohydrates and certain amino acids. For each turn of the TCA cycle, three molecules of NAD^+^ are reduced to NADH and one molecule of FAD is reduced to FADH_2_. These molecules then transfer their energy to the electron transport chain, which in turn releases energy so that it can be converted to adenosine triphosphate (ATP) as an energy source for bacteria [42]. Disruption of the metabolic activity of the bacterial TCA cycle causes the weakening of cellular respiration, making the energy supply inadequate, which can lead to cell death [43].

At the examined treatment concentration (MIC=10 mg ml^−1^) (Fig. 4a), *C. ternatea* anthocyanin fraction affected the TCA cycle, where it was found to have reduced the metabolite level of succinate (log_2_ FC=−1.28 and −3.8) at both 1 and 4 h, respectively, with a higher reduction at 4 h. The reduction of succinate by the anthocyanin fraction of *C. ternatea* may have disrupted the energy metabolism of the electron transfer chain by reducing the electron transfer to ubiquinone as the rate of the electron transfer from ubiquinone to NAD is limited by the low content of succinate, which serves as the main reductant of ubiquinone [41, 44].

In *E. coli *, ubiquinone is a lipid that plays an important role in the electron transfer chain to generate energy [45]. Upon treatment with the anthocyanin extract, the levels of NAD (log_2_ FC=−1.33 and −2.96) and ubiquinone-4 (log_2_ FC=−1.58 and −1.94) declined significantly at 1 and 4 h, respectively, with a higher reduction at 4 h (Fig. 4b). The metabolite levels of ubiquinone-6 (log_2_ FC=−2.88) and ubiquinol-8 (log_2_ FC=−1.61) were decreased at 4 h but not at 1 h. The anthocyanin fraction affected the TCA of *E. coli *, inducing a reduced level of its intermediate (succinate). This might have weakened its cellular respiration, reducing the energy supply, finally leading to cell growth inhibition [43].

The findings can be further supported by the effect of blueberry anthocyanins, which disrupted the bacterial TCA cycle and reduced the level of ATPase, impedeing respiratory metabolism. The blueberry anthocyanins could have acted by increasing the efflux of ATP from the cytoplasm of pathogens [38]. The production rate of formazan (to represent the metabolic activity of pathogens in the TCA cycle) gradually decreased with increasing concentration of blueberry anthocyanins to block the TCA of pathogens. The weakening of cellular respiration and inadequate energy supply may lead to cell death [38]. These findings show that the inhibition of energy production also plays an important role in the growth inhibition or killing of bacteria, as also reported in other studies [34, 46].

In conclusion, the anthocyanin fraction of *C. ternatea* has a potential bacteriostatic effect against *E. coli *. To the best of our knowledge, this is the first study to reveal significant metabolic perturbations of *C. ternatea* anthocyanin fraction against *E. coli *, revealing its mechanism of action. The anthocyanins in the fraction could have acted synergistically in exerting the bacteriostatic effect by perturbing glycerophospholipid metabolism, amino acid and energy metabolism, with these being the major pathways involved in the effect of *C. ternatea* anthocyanin fraction against *E. coli *.

Structure–activity relationship studies would be beneficial to understand the effect of the structural groups responsible for or contributing to the bioactivity. Future studies looking into combination treatment of *C. ternatea* anthocyanin fraction with clinical agents may be beneficial; its potential action as a bacteriostatic agent might enhance the antibacterial effect of clinical agents. Assessment of the impact on bacterial membrane integrity using imaging studies would be highly beneficial. It is also essential to carry out further studies to determine its effect on clinical and multidrug-resistant *E. coli* strains. The anthocyanin fraction could potentially be used to reduce *E. coli *-related infections. However, further studies investigating toxicity in pre-clinical models are also essential. This study provides novel metabolomic information on the mechanism of action of *C. ternatea* anthocyanin fraction to reduce *E. coli-*related infections.
